# Comparison of distance covered, physiological cost, and perceived exertion in four six-minute walk test protocols

**DOI:** 10.3389/fphys.2024.1395855

**Published:** 2024-05-30

**Authors:** Rodrigo Muñoz-Cofré, Mariano del Sol, Pablo A. Lizana, Alejandro Gómez-Bruton, María José Fuentes Andaur, Erika Soto Fierro, Gabriela Osorio Gonzalez, Paul Medina-González, Fernando Valenzuela-Aedo, Máximo Escobar-Cabello

**Affiliations:** ^1^ Programa de Doctorado en Ciencias Morfologicas, Universidad de la Frontera, Temuco, Chile; ^2^ Laboratory of Epidemiology and Morphological Sciences, Instituto de Biologia, Pontificia Universidad Catolica de Valparaiso, Valparaiso, Chile; ^3^ Centro de Investigación Interdisciplinaria en Biomedicina, Biotecnología y Bienestar (C3B), Pontificia Universidad Católica de Valparaíso, Valparaíso, Chile; ^4^ Exer-GENUD (EXERCISE-Growth, Exercise, Nutrition and Development Research Group), Faculty of Health and Sport Sciences, Department of Physiatry and Nursing, University of Zaragoza, Zaragoza, Spain; ^5^ Escuela de Kinesiología, Universidad Católica del Maule, Talca, Chile; ^6^ Departamento de Kinesiología, Facultad de Ciencias de la Salud, Universidad Católica del Maule, Talca, Chile

**Keywords:** walk test, exercise test, respiratory function tests, physical endurance, physical exertion

## Abstract

**Objective:** There is evidence that indicates that the Walked Distance (WD) in the 6-Minute Walk Test (6MWT) would be sensitive to the type of track and encouragement. The aim of study was compared the impact of track type and verbal encouragement provided in the 6MWT on WD, physiological cost, perceived exertion, and gait efficiency in healthy young adults unfamiliar with the test.

**Method:** WD, heart rate, subjective sensation of dyspnea (SSD), and fatigue (SSF) were measured in four 6MWT protocols: i) 30 m linear track and protocolized encouragement (LT + PE), ii) 30 m linear track and constant encouragement (LT + CE), iii) 81 m elliptical track and protocolized encouragement (ET + PE), and iv) 81 m elliptical track and constant encouragement (ET + CE). In addition, the Gait Efficiency Index (GIE) associated with physiological cost, dyspnea and fatigue was calculated and compared between the different protocols.

**Results:** The WD was significantly higher in the ET + CE protocol. The percentage of the heart rate reserve used (%HRRu) at minute 6 was higher in the ET + CE protocol. The SSD and SSD had difference in startup time between the protocols. The GEI was higher in %HRRu, SSD, and SSF for the ET + CE protocol.

**Conclusion:** The ET + CE protocol showed a significant increase in WD during the 6MWT in healthy young adults. Although it obtained the highest physiological cost, it did not present perceptual differences when entering cardiopulmonary assessment windows relevant to a more efficient test for the participant. It is advisable to discuss, based on the findings, the fundamental objective of the 6MWT and national and international recommendations to achieve a result as close as possible to the real maximal effort.

## 1 Introduction

The 6-Minute Walk Test (6MWT) has been studied extensively since the 1960s. Since then, it has developed significantly due to the evolution of disciplines that share the 6MWT as an aerobic capacity assessment test (Rabinovich et al., 2004). Its use ranges from diagnosing functional capacity in healthy individuals to predicting morbidity and mortality in subjects with respiratory system dysfunctions (McGavin et al., 1976). In 2002, the American Thoracic Society (ATS) published its practical guide to standardize the application of the 6MWT (ATS Committee on Proficiency Standards for Clinical Pulmonary Function Laboratories, 2002). In 2014 the ATS joint efforts with the European Respiratory Society to develop a systematic review and a technical standard to clarify the use and applications of the 6MWT (Holland et al., 2014; Singh et al., 2014). In Chile, also intending to standardize the 6MWT, the Chilean Society of Respiratory Diseases (Gutiérrez et al., 2009) published the 6MWT procedure manual. However, large differences continue to be observed in the relationship of 6MWD to clinical outcomes, the reference equations for 6MWD, instructions, stimulus, track length, and course location and design.

Despite these efforts, the evaluators who apply the 6MWT have not been able to unify their criteria, and the literature reports a significant diversity in the evaluation of the 6MWT, which makes it difficult to compare outcomes. In this sense, a growing number of studies are comparing different ways of executing the 6MWT. Analyzing respiratory rehabilitation centers in Latin America and the Iberian Peninsula, Tramontini et al. (2005) determined that more than 90% of the institutions performed the 6MWT with track of different distances (between 17 and 90 m) and different time data and verbal encouragement. In terms of the space layout, Sciurba et al. (2003), in their multicenter study of the 6MWT in patients with emphysema, found that the subjects walked on average 33 m more when performing the test on a circular track. By contrast, straight tracks of 15 and 50 m showed no significant differences between them.

Verbal encouragement has also been shown to affect the performance of subjects who do the 6MWT. Guyatt et al. (1984) determined that using phrases at regular intervals leads to an increase in 6MWT distance. Although the recommendation indicates recording physiological and perceptual parameters at the beginning and end of the test, different studies currently agree on the usefulness of minute-by-minute monitoring. In this context, in 2001, Escobar et al. reported the temporal control of physiological and perceptual variables with constant encouragement during the 6 minutes of the test in healthy children. The researchers concluded that this methodology showed a greater walked distance (WD) than the standardized encouragement every 1 minute. Therefore, the type and frequency of the encouragement might also be relevant when analyzing the performance and physiological cost of the 6MWT.

Human gait presents a pattern of successive and rhythmic strides that depend on an energy reserve for motor expression (Farris and Sawicki, 2012). In this context, energy optimization according to speed, whether walking or running, is fundamental (Saibene and Minetti, 2003). In this sense, the performance in meters obtained in the 6MWT acts synergistically with the physiological cost and perceived fatigue to adjust the level of gait efficiency. Muñoz et al. (2016) highlighted the need to balance the integration between perceived exertion, physiological cost, and performance to control variability in the 6MWT. Thus, considering the layout of the physical space, verbal encouragement, and the variables to be recorded is essential to standardize the 6MWT.

Therefore, this study aimed to compare the impact of track type and verbal encouragement provided in the 6MWT on WD, physiological cost, perceived exertion, and gait efficiency in healthy young adults unfamiliar with the test.

## 2 Materials and methods

### 2.1 Participants

**FIGURE 1:**
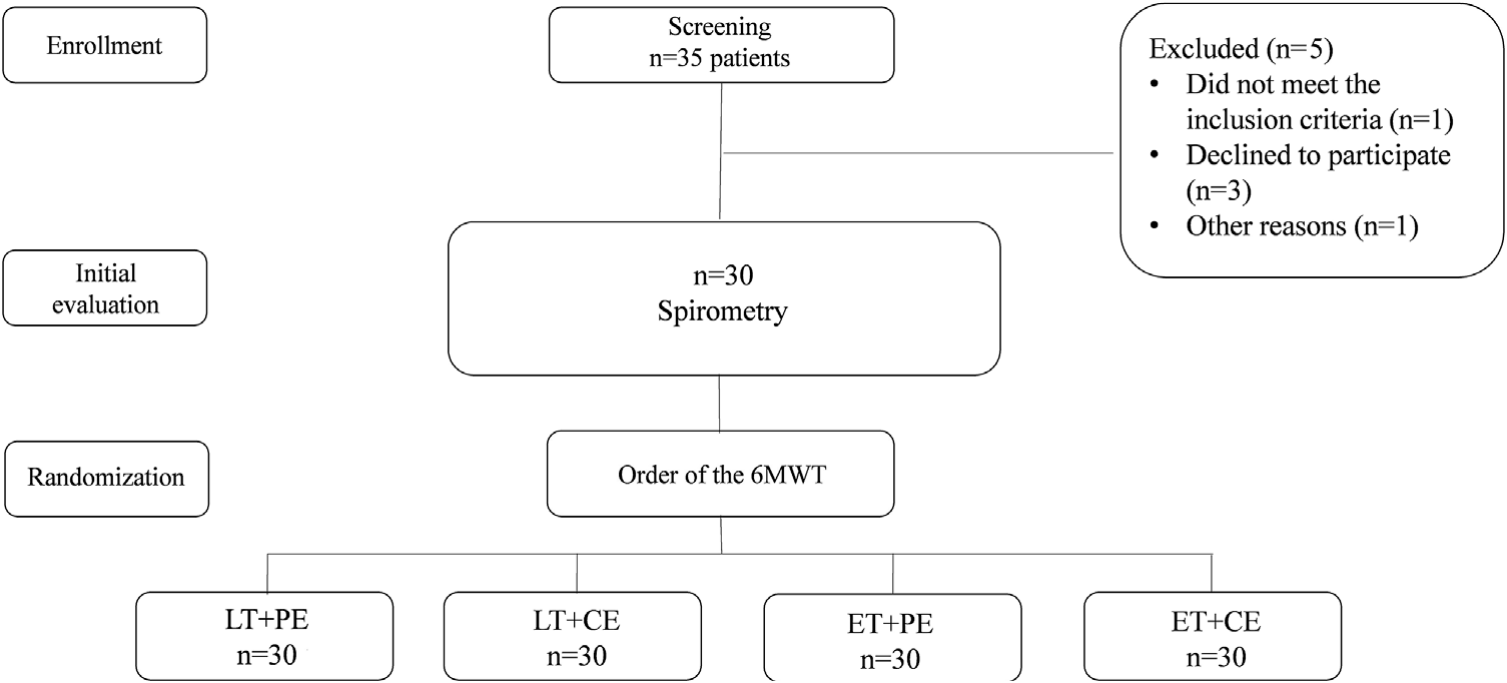
Flow chart of the participant selection. 6MWT: 6-min walk test; LT + PE: linear track plus protocolized encouragement; LT + CE: linear track plus constant encouragement; ET + PE: elliptical track plus protocolized encouragement; ET + CE: elliptical track plus constant encouragement.

A cross-sectional comparative study was conducted between December 2016 and April 2017. The sample size was calculated from the total population of 4,839 university students through the sample size calculation software Ene 3.0^®^. With a significance level of 0.05, a statistical power of 80%, a dropout rate of 10%, a mean of 820 m, and a standard deviation of 12 m in the WD (Muñoz et al., 2016), the sample calculation was 32 students from 18 to 25 years of age. The inclusion criteria were 1) no history of morbid conditions (diabetes, heart problems, asthma, etc.), 2) a body mass index (BMI) between 18.5 and 24.9 kg/m^2^, 3) a forced expiratory volume in the first second (FEV_1_) >80% of the predicted value, 4) no cognitive alterations that impeded performing the test, and 5) unfamiliarity with the 6MWT (Figure 1). The study was explained orally to each participant, and then if they decided to participate volunteers read and signed the informed consent.

### 2.2 Forced vital capacity

Forced vital capacity was measured in a plethysmograph (Platinum Elite Model DL^®^, St. Paul, Minnesota, United States). The subjects ventilated to tidal volume through the pneumotachograph for five respiratory cycles, and they were instructed to take a maximal inspiratory maneuver and then a maximal forced expiration. The best test of a minimum of three acceptable and reproducible maneuvers was selected (Graham et al., 2019).

### 2.3 6MWT protocols

The 6MWTs were performed on an 18 m × 38 m concrete surface, with no roof, at 09:00 a.m. (approximately 18°C), one test per day, on four consecutive days. Four 6MWT protocols were applied: 1) 30 m linear track and protocolized encouragement (LT + PE) (ATS Committee on Proficiency Standards for Clinical Pulmonary Function Laboratories, 2002), 2) 30 m linear track and constant encouragement: It consisted of repeating the following phrase every 15 s: “You are doing well. Keep up the good work” (LT + CE), 3) 81 m elliptical track and protocolized encouragement (ET + PE), and 4) 81 m elliptical track and constant encouragement (ET + CE) (Muñoz et al., 2015). Three evaluators participated in applying the 6MWT: The first evaluator delivered instructions and encouragements depending on the protocol. The second one was in charge of time and recorded the data, and the third evaluator recorded the number of turns and WD. All participants performed the 6MWT with the four proposed protocols. The order of the protocols was distributed through a probability mode without replacement (Figure 1).

In addition, supine heart rate (SHR) was measured in all participants after five minutes of rest (Medina et al., 2015). During the 6MWT, the modified Borg (1982) scale was used to quantify the subjective sensation of dyspnea (SSD) and fatigue (SSF) (Fletcher et al., 2001). A heart rate monitor (Polar®FS3, Kempele, Finland) monitored the working heart rate (Fletcher et al., 2001) from minutes zero to six (WHR6MWT). In the three minutes after completion of the 6MWT, recovery was recorded in a standing position. The percentage of heart rate reserve used (%HRRu) was obtained from the following formula (Medina et al., 2015): %HRRu = [100 x (WHR6MWT-SHR)]/[(220-age)- SHR].

The data recorded were WD in m, physiological cost in %HRRu, and SSD and SSF in values of 0/10.

### 2.4 Gait efficiency index

The analysis of the physiological cost of walking and transportation by Medina et al. (2015) was used to determine gait efficiency. This Gait Efficiency Index (GEI) was obtained by applying the following formulas: GEI and physiological cost: % (oWD-rWD)/Physiological cost, GEI, and dyspnea: % (oWD-rWD)/SSDx100, GEI, and fatigue: % (oWD-rWD)/SSFx100.

The numerator is the percentage difference between the obtained value during the 6MWT (oWD) minus the reference value (rWD), according to Osses et al. (2010). In the denominator, the %HRRu at minute 6 was used for physiological cost, the value of the modified Borg scale of dyspnea and fatigue for perception at minute 6 (x100). The higher the index presents positive values, the greater the efficiency (for the same performance, less consumption of the energy reserve would be required).

### 2.5 Statistical analysis

The data are presented as means ± SD unless otherwise stated. The Shapiro-Wilk test was used to determine data normality. The comparison of physiological variables and ventilatory function between genders was compared using Studen’s t tests for unpaired samples or Mann-Whitney U. The variables obtained during the 6MWT were analyzed by comparing minute to minute (from minute 0 to 6 corresponding to the test and from minute 7 to 9 corresponding to the recovery phase). The ANOVA test repeated measures o Friedman were used to compare WD, HR, SSF, and SSD among protocols, depending on the data distribution. Significant differences were considered significant when *p* < 0.05. The statistical analysis was performed with GraphPad Prism (version 5.0^®^, San Diego, United States).

## 3 Results

A total of 35 subjects were recruited; one did not meet the inclusion criteria, three declined to participate and one for other reasons (Figure 1). The distribution by gender was 11 women and 19 men, the mean age was close to 19.10 ± 2.17 years, and the basic physiological variables and ventilatory function were within normal parameters. The general characteristics of the participants are shown in Table 1.

**TABLE 1:** General characteristics of the sample.

Variables	Total sample	Male (n = 19)	Female (n = 11)	*p*-value
Age (years)	19.10 ± 2.17	19.05 ± 2.04	19.18 ± 2.48	0.158^MW^
Weight (kg)	63.06 ± 8.61	67.84 ± 6.49	54.82 ± 4.62	0.0001^MW^
Height (m)	1.66 ± 0.08	1.72 ± 3.59	1.56 ± 4.3	0.0001^MW^
BMI(kg/m^2^)	22.73 ± 2.18	22.91 ± 2.30	22.42 ± 2.03	0.279^MW^
HR (bpm)	75.10 ± 6.90	73.21 ± 7.35	78.36 ± 4.74	0.188^MW^
RR (cpm)	18.03 ± 1.44	17.95 ± 1.51	18.18 ± 1.40	0.254^t^
SBP (mmHg)	112.64 ± 4.52	113.95 ± 4.43	110.39 ± 3.88	0.156^t^
DBP (mmHg)	66.99 ± 6.07	67.11 ± 5.73	66.80 ± 6.90	0.335^t^
MAP (mmHg)	82.00 ± 4.94	83.00 ± 4.69	81.00 ± 5.47	0.265^t^
FVC (L)	4.68 ± 0.99	5.33 ± 0.56	3.56 ± 0.28	0.0001^t^
Predicted FVC (%)	111.13 ± 10.49	113.42 ± 11.27	107.18 ± 7.96	-
FEV_1_ (L)	4.02 ± 0.76	4.46 ± 0.60	3.26 ± 0.18	0.0001^MW^
Predicted FEV_1_ (%)	110.20 ± 12.37	110.32 ± 14.79	110.00 ± 7.07	-
FEF 25%–75% (L/s)	4.45 ± 1.15	4.65 ± 1.36	4.10 ± 0.52	0.182^t^
Predicted FEF 25%–75% (%)	105.43 ± 26.72	102.95 ± 30.59	109.28 ± 18.80	-
FEF max (L/s)	8.52 ± 1.63	9.35 ± 1.43	7.09 ± 0.69	<0.001^t^
Predicted FEF max (%)	109.50 ± 16.74	109.37 ± 18.09	109.28 ± 14.95	-

Vital sign measurements were performed in the supine position and after resting for 5 min in this position. n: number of subjects; bpm: beats per minute; Kg: kilograms; m: meters; BMI: body mass index; kg/m^2^: kilograms/square meters; HR: heart rate; bpm: beats per minute; RR: respiratory rate; cpm: cycles per minute; SBP: systolic blood pressure; mmHg: millimeters of mercury; DBP: diastolic blood pressure; MAP: mean arterial pressure; FVC: forced vital capacity; L: liters; FEV_1_: forced expiratory volume in the first second; FEF, 25%–75%: forced expiratory flow between 25% and 75% of vital capacity; L/s: liters per second; FEFmax: maximum forced expiratory flow; m: meters; t: t de Student; MW: U Mann-Whitney.

**FIGURE 2:**
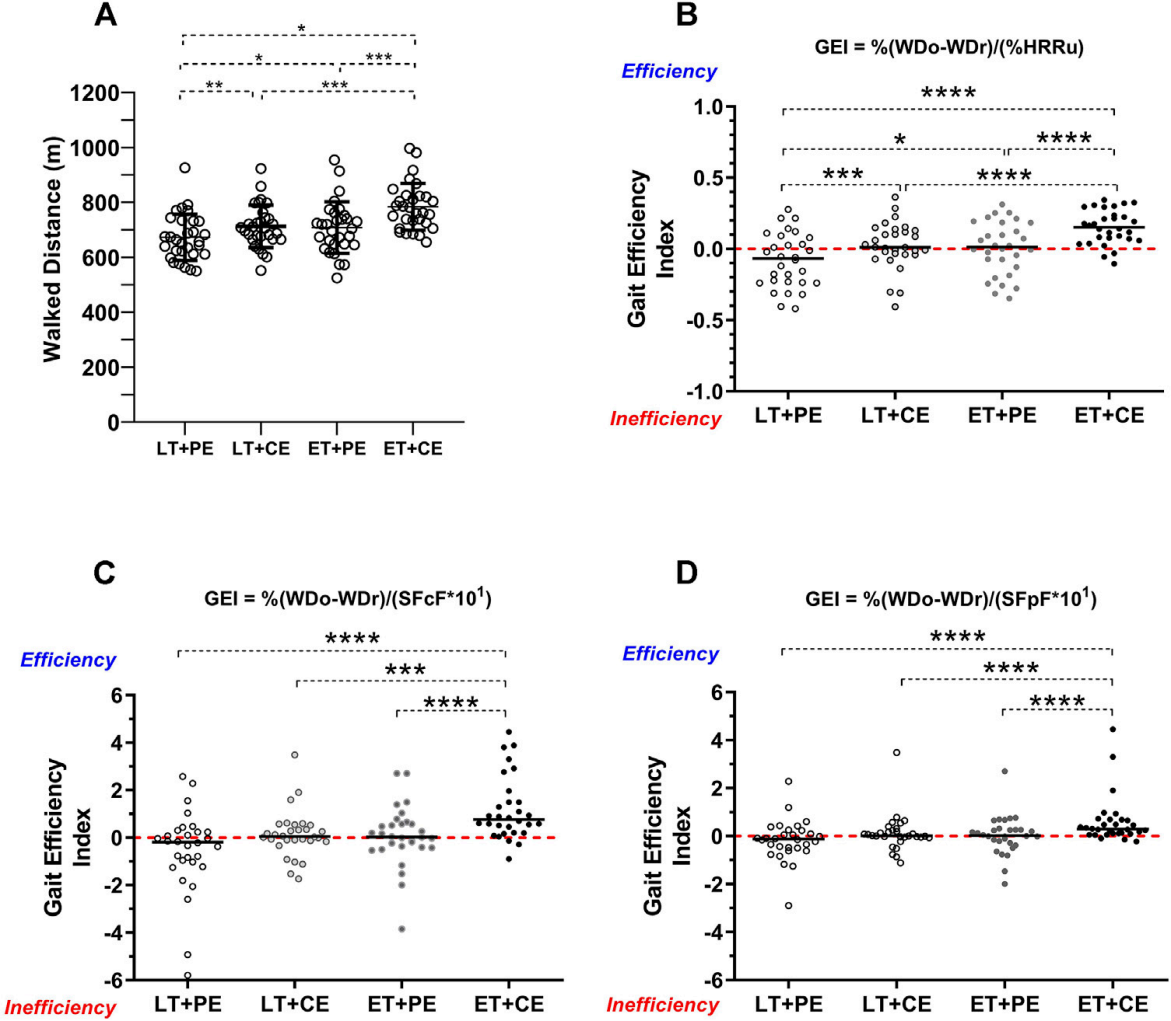
Walked distance and gait efficiency of the study sample. LT + PE: linear track plus protocolized encouragement; LT + CE: linear track plus constant encouragement; ET + PE: elliptical track plus protocolized encouragement; ET + CE: elliptical track plus constant encouragement; GEI: gait efficiency index; oWD: obtained walked distance; rWD: reference walked distance; %HRRu: percentage of heart rate reserve used; **(A)** walked distance according to tracks and verbal encouragement; **(B)** comparison of gait efficiency index according to the percentage of heart rate reserve used; **(C)** comparison of gait efficiency index according to subjective sensation of dyspnea; **(D)** comparison of gait efficiency index according to subjective sensation of fatigue; *: *p* < 0.05; **: *p* < 0.001; ***: *p* < 0.001; ****: *p* < 0.0001. Statistical tests used: Kruskal–Wallis and *post hoc* Dunn.

Concerning the WD the ET + CE protocol (784 ± 85 m) was significantly higher than ET + PE (708 ± 94 m), LT + CE (713 ± 68 m) and LT + PE (672 ± 84 m) (Figure 2A). The temporal control of %HRRu, was significantly higher at minute 6 in the ET + CE test in relation to the LT + PE test (Table 2). On the other hand, dyspnea, and fatigue on the test showed no significant differences among the different protocols (Table 3).

**TABLE 2:** Temporal control of percentage of heart rate reserve used according to the implementation protocol of the 6MWT in asymptomatic and healthy university students in Talca, Chile.

Type	Time	LT + PE	LT + CE	ET + PE	ET + CE	*p-value*
Standing	M0	10.6 ± 7.8 [7.7–13.5]	9.9 ± 8.6 [6.8–13.2]	11.6 ± 7.8 [8.7–14.6]	12.6 ± 10.6 [8.6–16.5]	*0.682*
Walking Test	M1	41.5 ± 15.3 [35.8–47.2]	49.5 ± 14.3 [44.2–54.9]	42.7 ± 15.7 [36.9–48.6]	45.1 ± 17.8 [38.5–51.8]	*0.222*
	M2	46.8 ± 16.8 [40.6–53.1]	54.6 ± 15.8 [48.7–60.5]	49.4 ± 16.2 [43.4–55.4]	54.1 ± 14.7 [48.6–59.5]	*0.179*
	M3	49.4 ± 17.3 [42.9–55.8]	55.9 ± 16.2 [49.9–62.0]	53.7 ± 13.5 [48.7–58.8]	59.4 ± 13.9 [54.3–64.6]	*0.084*
	M4	52.6 ± 17.1 [46.2–55.9]	58.5 ± 16.1 [52.5–64.5]	55.9 ± 15.1 [50.3–61.6]	60.9 ± 14.4 [55.5–66.3]	*0.207*
	M5	54.8 ± 17.9 [48.1–61.5]	61.7 ± 15.2 [55.9–67.4]	58.2 ± 16.9 [51.8–64.5]	64.4 ± 13.6 [59.4–69.5]	*0.352*
	M6	58.5 ± 15.9 [52.5–64.4]	66.6 ± 13.5 [61.6–71.6]	60.1 ± 16.0 [54.2–66.1]	69.8 ± 12.7* [65.0–74.5]	*0.009*
Recovery	M7	29.7 ± 13.0 [24.8–34.6]	33.9 ± 17.3 [27.4–40.4]	31.3 ± 15.2 [25.6–37.0]	37.7 ± 17.1 [31.3–44.0]	*0.228*
	M8	25.6 ± 11.9 [21.2–30.1]	26.0 ± 14.6 [20.5–31.4]	25.6 ± 12.8 [20.8–30.3]	28.3 ± 14.2 [23.0–33.6]	*0.838*
	M9	19.8 ± 8.3 [16.7–22.9]	21.8 ± 13.4 [16.8–26.8]	23.1 ± 11.1 [18.9–27.2]	23.6 ± 12.1 [19.0–28.1]	*0.589*

Mean ± standard deviation and 95% confidence intervals for the temporal behavior of the percentage of heart rate reserve according to the performance protocols for the 6MWT. M: minute; LT + PE: linear track plus protocolized encouragement; LT + CE: linear track plus constant encouragement; ET + PE: elliptical track plus protocolized encouragement; ET + CE: elliptical track plus constant encouragement. The inter-subject *p*-value considers the general comparison for every minute of the test among the different implementation protocols. Friedman test was used. Italic values represent the *p < 0.05, LT + PE, vs. ET + CE.

**TABLE 3:** Temporal control of the SSF and SSD according to the implementation protocol of the 6MWT in asymptomatic and healthy university students in Talca, Chile.

		Subjective sensation of fatigue					Subjective sensation of dyspnea				
Type	Time	LT + PE	LT + CE	ET + PE	ET + CE	*p-value*	LT + PE	LT + CE	ET + PE	ET + CE	*p-value*
Standing	M0	0 [0–1]	0 [0–1]	0 [0–1]	0 [0–1]	*0.190*	0 [0–1]	0 [0–1]	0 [0–1]	0 [0–1]	*0.112*
Walking Test	M1	0 [0–2]	0 [0–4]	0 [0–3]	0 [0–3]	*0.511*	0 [0–2]	0 [0–4]	0 [0–3]	0 [0–3]	*0.060*
	M2	0 [0–3]	1 [0–6]	0 [0–4]	0 [0–3]	*0.467*	0 [0–3]	1 [0–5]	0 [0–4]	0 [0–3]	*0.078*
	M3	1 [0–4]	1 [0–5]	1 [0–3]	1 [0–4]	*0.311*	1 [0–4]	1 [0–5]	1 [0–4]	0 [0–4]	*0.128*
	M4	1 [0–4]	1 [0–5]	1 [0–4]	1 [0–4]	*0.893*	1 [0–4]	1 [0–5]	1 [0–4]	1 [0–4]	*0.566*
	M5	1 [0–5]	1 [0–4]	1,5 [0–5]	1 [0–5]	*0.721*	1 [0–5]	1 [0–6]	2 [0–5]	1 [0–5]	*0.266*
	M6	1 [0–5]	2 [0–6]	1,5 [0–5]	1 [0–5]	*0.559*	1 [0–5]	2 [0–6]	2 [0–5]	1 [0–5]	*0.156*
Recovery	M7	0 [0–3]	1 [0–4]	0 [0–3]	0 [0–3]	*0.248*	0 [0–3]	1 [0–4]	1 [0–3]	0 [0–3]	*0.100*
	M8	0 [0–3]	0 [0–3]	0 [0–2]	0 [0–2]	*0.829*	0 [0–2]	0 [0–3]	0 [0–2]	0 [0–2]	*0.507*
	M9	0 [0–2]	0 [0–2]	0 [0–2]	0 [0–2]	*0.883*	0 [0–2]	0 [0–2]	0 [0–2]	0 [0–2]	*0.697*

Median and minimum-maximum values in parenthesis of the temporal behavior of the subjective sensation of fatigue on a scale from 0 to 10 according to the 6MWT, implementation protocols. LT + PE: linear track plus protocolized encouragement; LT + CE: linear track plus constant encouragement; ET + PE: elliptical track plus protocolized encouragement; ET + CE: elliptical track plus constant encouragement. The inter-subject *p*-value considers the general comparison for every minute of the test among the different implementation protocols. Friedman test was used.

The results of the GEI are presented in Figure 2. The ET + CE protocol delivered significantly higher efficiency for %HRRu (Figure 2B), SSD (Figure 2C), and SSF (Figure 2D) compared to the linear circuits. The LT + PE protocol showed a negative and significantly lower GEI than the ET + CE protocol in the variables %HRRu, SSD, and SSF. In summary, it could be inferred that the ET + CE protocol has higher performance in WD with less energy reserve consumption.

## 4 Discussion

The current study observed that regulated verbal encouragement and the track where the 6MWT is performed significantly affect the performance in meters, physiological cost, and perceived exertion. There is a greater performance in meters with the ET + CE with a similar or lower transportation cost than the other protocols (Figure 2; Tables 2, 3). Thus, the elliptical track and the constant encouragement allow for a gait with more efficient characteristics (Medina et al., 2015) (Figures 2A–C).

### 4.1 Distance covered

The results indicate a significant increase in the WD of the ET + CE protocol compared to the LT + PE, LT + CE, and ET + PE. This coincides with the report by Sciurba et al. (2003), who observed that the WD on continuous oval tracks was greater by 92 feet (≈28 m) compared to straight tracks. Bansal et al. (2008) reported a difference of 13.17 m more in performance in subjects who performed the test on a continuous rather than a straight track. In addition, Muñoz et al. (2015) evaluated the impact of the track on the performance of the 6MWT in university students, comparing two tracks—one straight of 30 m and another elliptical of 400 m—finding that participants walking in the elliptical track performed a higher WD than when walking on the linear track (809.0 ± 8.7 m vs. 764.0 ± 12.2 m; *p* = 0.034). These results are consistent with the results reported in this study, where the WD was higher on the elliptical track.

The differences in WD may be due to constant accelerations and decelerations in gait not occurring on the elliptical track, which are present on a straight track with a delimited length. In this respect, Medina et al. (2016) proposed that gait be assessed in older adults on an elliptical track because this track would allow a gait pattern that would better resemble natural conditions, as opposed to a straight unidirectional track, where the displacement would be conditioned by turns. In this sense, Pinochet et al. Indicate that the changes in direction that the subject undergoes during the turns on the one-way track cause the walking speed to decrease. At the same time, vision would be a distracting factor on a straight track because, for a few seconds, the subject focuses on the endpoint of the track and the subsequent turn, neglecting the objective of the test, which is to walk as fast as possible (Pinochet et al., 2003). Therefore, these three factors (vision, changes of direction, and loss of the test objective) would decrease the mechanical efficiency of walking on a linear track compared to an elliptical one, a situation supported by the results obtained in this study (Figure 2A).

Continuous verbal encouragement improved the WD, which is observed when contrasting the constant encouragement protocol (ET + CE and LT + CE) with the protocolized protocol (ET + PE and LT + PE). Verbal encouragement motivates people to increase their commitment and reach real exertion in maximal effort tests, recommended in several protocols (Cabillic et al., 2011; Marinho et al., 2014) and ATS regulations (Holland et al., 2014; Marinho et al., 2014; Singh et al., 2014). Finally, from the cardio-metabolic point of view, it provokes a more effective physiological response in accordance with the expected workload for physical performance tests in indicators such as VO2max and maximal HR (Midgley et al., 2017).

### 4.2 Physiological cost

The %HRRu exhibited a rapid rise between standing (minute 0) and minute 1 and then followed the same but less pronounced pattern, which was similar for all the protocols. This agrees with the report by Escobar et al. (2001), who performed the 6MWT with continuous encouragement in healthy Chilean children. Muñoz et al. (2015) and Baeza et al. (2014), in university students and patients with chronic obstructive pulmonary disease (COPD), respectively, observed the same pattern. In the recovery phase, once the test was over, an abrupt drop in HR was observed, which did not reach the baseline shown in standing. This is supported by what was stated by Ostojic et al. (2010), who indicated that HR recovery during the first minute after work is slower after high-intensity exercise because the reactivation of the parasympathetic system is delayed through the vagus nerve.

The highest %HRRu values occur in the final minute of the test for the ET + CE protocol (Table 2), with no significant differences in the SSF and SSD. Parallel to this phenomenon, the highest WD was reached. These factors are consistent with greater walking efficiency in the ET + CE protocol (Figure 2B). In this context, The ET + CE group was closer to reaching the real maximal exertion.

### 4.3 Perceived dyspnea and fatigue

The SSD and SSD had difference in startup time between the protocols (Table 3). The type of track used in the 6MWT seems to be the factor that makes the difference to the SSF because, in the protocols with linear track (LT + PE-LT + CE), the SSF appears earlier than those with elliptical track. In this context, muscle recruitment during speed changes would play a fundamental role in this track. In this respect, Gandevia (2001) indicates that muscle fatigue, although it resides in the brain, originates in muscle fibers. In this context, the appearance of “peripheral fatigue” due to the turns made on the linear track would make it difficult to maintain a constant walking pace, ultimately impacting the WD. Thus, these indicators could be used to indirectly qualify the behavior of different mechanisms of exertion during walking at intensities and times greater than the walking required for daily life activities.

The appearance of SSF in the lower limbs after SSD could be illustrative of “better” or “worse” muscle resistance during aerobic exercise (Santos et al., 2020). The theory posits that the increase in respiratory rate due to the higher loads imposed by exercise gradually increases the expiratory reserve volume, causing a decrease in inspiratory capacity (Lutfi, 2017). In addition, it would alter the distribution of oxygen in the periphery, triggering skeletal muscle fatigue and resulting in a significant reduction in WD on the 6MWT (Figure 2A). This would explain the time of onset of SSD and SSF, which, although they did not present significant differences, were expressed in different ways. Continuous encouragement added to the linear track made the SSF appear before the SSD. This may be related to the faster recruitment of all the lower limb muscles and how the encouragement would achieve this phenomenon (Segers et al., 2007).

### 4.4 Gait efficiency index

Figure 2 represents the proposed GEI for the four protocols according to the behavior of %HRRu (Figure 2B), SSD (Figure 2C), and SSF (Figure 2D). The ET + CE protocol proved significantly more efficient in all three variables than the other 6MWT protocols (Figure 2B). This is consistent with reports for reaching the physiological (Bassett, 2002) and mechanical (Saibene and Minetti, 2003) steady state of optimal walking speed with the lowest possible consumption. Therefore, the results obtained in this study make it possible to infer the minimum requirements of space and encouragement to evaluate and intervene in the context of optimal levels of locomotion in human beings. This would explain the greater WD (Figure 2A) with no significant changes in perceived exertion (Table 3).

### 4.5 Interaction of physiological cost and perceived exertion

The variables physiological cost, SSF, and SSD on the 6MWT did not show any significant differences among the different protocols (Tables 2, 3). However, these parameters presented different behaviors according to the protocol applied. If we consider what was proposed by Muñoz et al. (2015) in relation to i) a %HRRu between 55% and 90% (corresponding to a VO2máx between 40% and 85% (Fletcher et al., 2001), ii) an SSD of 2–4 points (“light” to “somewhat heavy” exertion), and iii) SSF of 0.5–2 points (“very, very light” to “light” equivalent to a workload between 30% and 49% of the maximum voluntary contraction) (Borg, 1982), as control ranges in the execution of the 6MWT. In the protocols with protocolized encouragement (LC + PE; EC + PE), the %HRRu exceeds 55% at minutes 4 and 6. Moreover, the SSD reaches two points at minutes 2 and 3, respectively. Likewise, constant encouragement (LT + CE; ET + CE) made it possible to exceed 55% of %HRRu at minutes 3 and 2. Associated with this behavior, the SSD exceeded two points at minute two in both protocols (Figures 3B,D), respectively. This is consistent with Escobar et al. (2001), who pointed out that despite the correlation between the variables SSD and HR, subjective perceptions do not show a notable increase like HR. Finally, in the ET + CE protocol, entry into the control range in the SSD is earlier than in the SSF. In this regard, this behavior could explain, in part, the greater performance obtained with this modality since, first, it allows entry to cardiorespiratory overload (SSD), which has been reported to be better tolerated than peripheral overload (SSF) (Zghal et al., 2015). Thus, the test could be performed with a greater cardiopulmonary vs. musculoskeletal or neuromotor component, guaranteeing better tolerance in the muscle *in situ* for the 6MWT.

### 4.6 Limitations and projections

The limitations of the present study include the specificity of the sample studied. In such a scenario, the projections of these results first involve applying the ET + CE model in different age ranges and functional contexts to characterize the population with dysfunction. However, it has the strength of studying a homogeneous sample to observe changes in the different 6MWT protocols. On the other hand, the tools used to determine physiological cost and perceived fatigue do not present the maximum clinical rigor. In this context, we seek to improve the measurements by incorporating oxygen consumption as a reference indicator. On the other hand, an important projection is to explore in relation to the minimum necessary distance of the elliptical track, with the purpose of strengthening clinical applicability.

## 5 Conclusion

The results of this study make it possible to report that the protocol with ET + CE presented a significant increase in the WD during the 6MWT in healthy young adults unfamiliar with this test. This protocol obtained the greatest physiological cost and demonstrated no perceived differences in the entry to cardiopulmonary assessment windows relating to a test of greater efficiency for the participant. Given the differences found in this study, it is necessary to discuss the fundamental objective of the 6MWT and the national and international recommendations to obtain a result as close as possible to the true maximum exertion.

## Data Availability

The raw data supporting the conclusion of this article will be made available by the authors, without undue reservation.
